# Supplementary material for: Vitamin D and Diseases of Mineral Homeostasis: A Cyp24a1 R396W Humanized Preclinical Model of Infantile Hypercalcemia Type 1
Source: Nutrients. 2022 Aug 6;14(15):3221. doi: 10.3390/nu14153221 (PMC9370611; doi:10.3390/nu14153221)
Supplement: Supplementary file 1 [file nutrients-14-03221-s001.zip › nutrients-1854374-supplementary.pdf]

SUPPLEMENTARY FIGURES

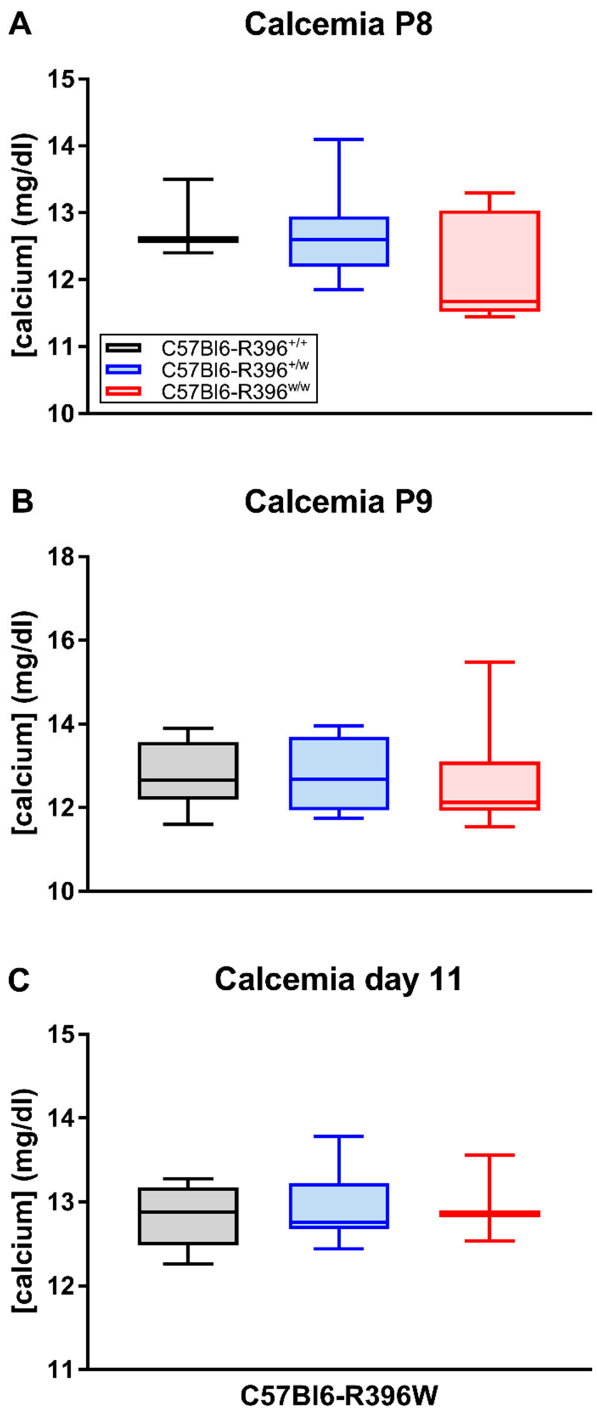

Supplementary Figure S1. Serum calcium levels in C57Bl6-R396W littermates. Total serum calcium was measured using an automated analyzer. A, postnatal day 8; B, postnatal day 9; C, postnatal day 11. No statistical differences by one-way ANOVA.

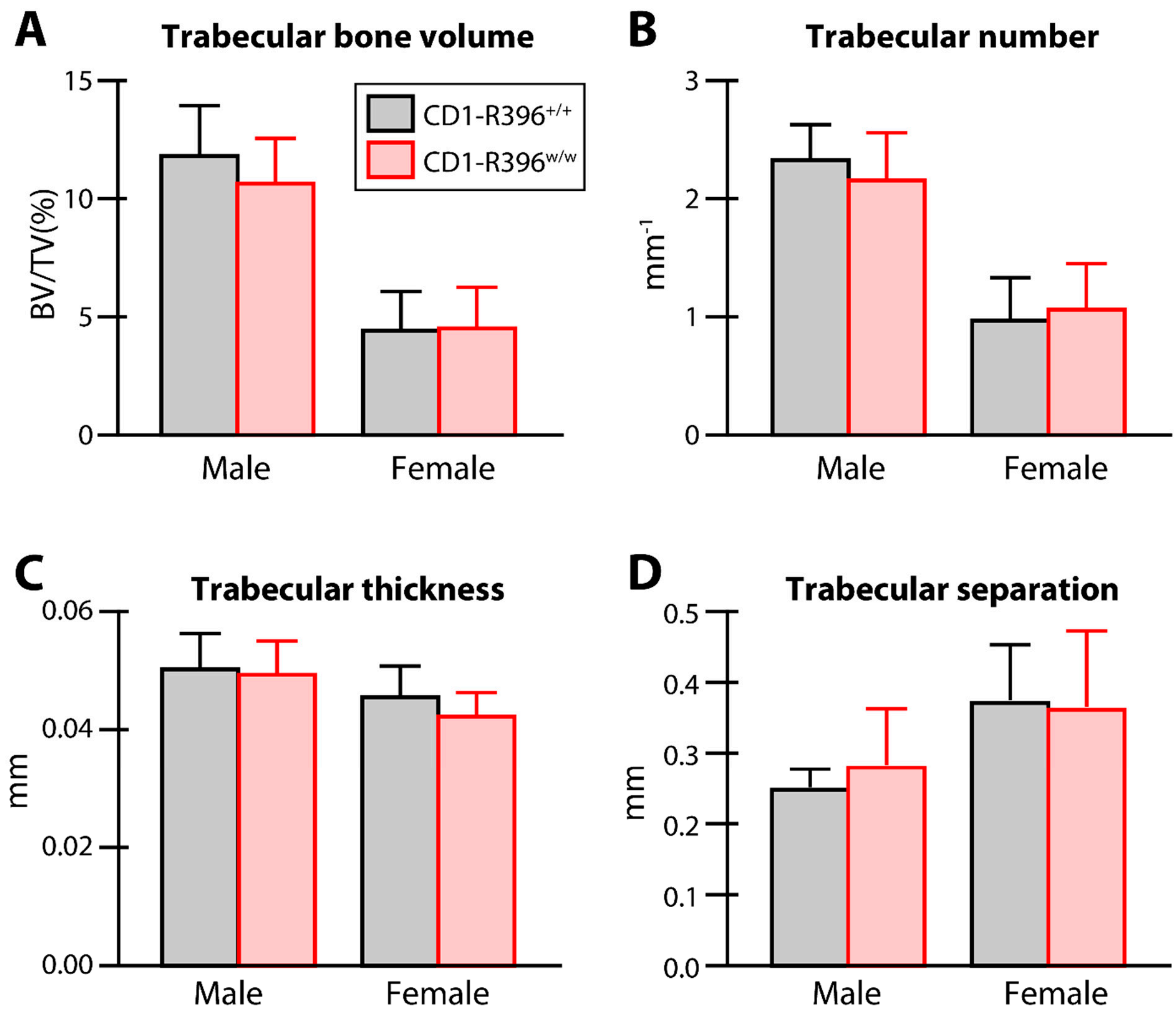

Supplementary Figure S2. Steady-state trabecular parameters in CD1-R396W knock-in strain. Tibiae from mutant and wild-type littermates of both sexes were analyzed by μCT at 3 months of age.

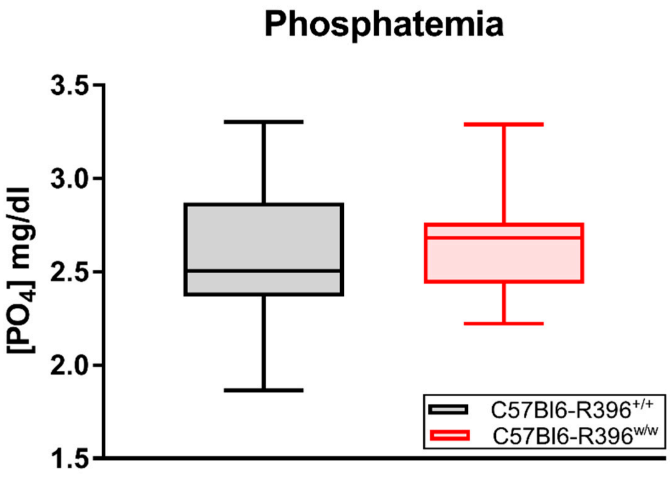

Supplementary Figure S3. Serum phosphate levels in surviving wild-type and mutant C57Bl6-R396W mice at 3 months of age.
